# Religious affiliation seldom seems to influence hiring or competence ratings of job applicants: studies conducted in Sweden and in the USA

**DOI:** 10.1186/s40359-022-00927-0

**Published:** 2022-09-19

**Authors:** Nathalie Hallin, Daniel Västfjäll, Gerhard Andersson

**Affiliations:** 1 Department of Behavioural Sciences and Learning, Division of Psychology, Linköping University, Linköping, Sweden; 2 Department of Clinical Neuroscience, Karolinska Institutet, Psychiatry unit, Stockholm, Sweden

**Keywords:** Religion, Atheism, Ingroup favouritism, Outgroup derogation, Recruitment

## Abstract

**Background:**

Religion is an important ingroup characteristic for many people. For different reasons, people with different religious affiliations might prefer members of their religious outgroup. Previous studies have investigated perceptions of and behaviour toward religious ingroup and outgroup members in various contexts. The four studies presented here investigated whether competence and likeability ratings differ depending on the target’s and participant’s religious affiliations in a recruitment context. Two studies were conducted in Sweden, while the other two were conducted in the USA.

**Methods:**

Participants in 4 studies rated a Christian, Muslim or atheist job applicant and a control applicant on 4 competence and 3 likeability items on 7-point Likert scales. The difference in ratings between the target applicant and control applicant was used to measure perceived competence and likeability of the target applicant. In the two latter studies, one in Sweden and one in the USA, participants also chose to hire either the target or the control applicant.

**Results:**

Overall, participants in three studies rated control applicants as more likeable than target applicants. In the two US studies, targets were also rated as less competent than control applicants. Christian participants in the two US studies rated the Christian applicant as more likeable than both other targets. In the second US study, atheist participants rated Christians as less likeable than both other targets. In one of the Swedish studies, atheist participants rated the atheist applicant as more likeable than both other targets. The only significant difference in competence ratings between targets was made by Christian Swedes, who rated Muslim applicants as less competent than Christian applicants. The only significant difference in hiring decisions was that Swedish atheist participants hired Christians less often than they hired control applicants.

**Conclusion:**

Together, the results suggest that job applicants are sometimes viewed as more likeable if they belong to a religious ingroup rather than a religious outgroup, but that this only rarely translate to significant differences in competence ratings or hiring decisions.

**Supplementary Information:**

The online version contains supplementary material available at 10.1186/s40359-022-00927-0.

## Introduction

People tend to favour ingroup members over outgroup members in many ways. For instance, ingroup members are regarded more positively, rewarded more, and favoured when it comes to cooperation. Ingroups can be groups that are important in people’s lives, such as religious groups or organisations, but can also be created arbitrarily in experimental studies [1]. In a meta-analysis, Balliet et al. [1] found that people cooperated more with ingroup members than with outgroup members, but cooperation did not differ between outgroup members and uncategorised strangers. Thus, in contexts where people are asked to cooperate, ingroup favouritism seems to drive the discrimination rather than outgroup derogation [1].

Religion is an important ingroup characteristic for many people and according to a theory on the function of religion, religions have evolved culturally to facilitate cooperation [2, 3]. Norenzayan et al. [3] argued that religions with moralising gods evolved culturally because of their ability to create cooperation within a larger group. These gods served as a substitute for reputation, which was an effective tool for small-group cooperation, but the role of reputation diminished as societies grew past the point of each member being able to keep track of reputations of all other members in the group. The existence of moralising gods who had the ability to monitor all human behaviour and could punish immoral actions would serve as an incentive to adhere to moral norms and enable greater cooperation. Over time, these religions out-competed other types of religions in societies, either because increased prosperity made these societies grow when competing societies failed – or because competing societies adopted the successful religions [3].

According to the abovementioned theory, religious people may distrust nonbelievers, as they are expected to lack the religious incentive to adhere to moral rules. In line with this reasoning, several studies have found that atheists in some settings are distrusted by people in general [4–10]. In addition to the possible outgroup derogation, religious people have been found to be more prosocial towards people who share their religious beliefs [11].

It is also possible that nonbelievers are less cooperative towards religious people. Although nonreligious people have been found to be generally *less* prejudiced, they seem to be *more* prejudiced towards people who might be a threat to their own worldviews [12]. In a study by Uzarevic et al. [13] European nonbelievers were willing to help a religious person to the same extent as a nonreligious person with a neutral cause, but were less willing to help a religious person with a religious cause. In addition, French atheists were less willing to help a religious person even with a neutral cause [13].

Although there are studies on religious ingroup favouritism and outgroup derogation [14–16], most studies have been conducted in relatively religious countries. The factors that influence perceptions of religious ingroup and outgroup members might differ or operate differently in highly secular countries. In Sweden, few people consider religion to be an important part of their life and a majority do not believe in God [17]. Thus, religious values are unlikely to be perceived as a major threat to nonreligious people in everyday life. Furthermore, the general level of trust is high in Sweden [17], despite the low religiosity, which means that other factors are likely to contribute to people trusting each other. This suggests the possibility that atheists are less distrusted by religious people in Sweden.

A series of studies by Gervais and colleagues have suggested that people in the USA view immoral acts like murder as more representative of atheists—even when participants themselves do not believe in God [5, 6, 8]. Cook et al. [18] conducted two experiments with American students and found that atheists were associated with moral disgust, but not with physical disgust. Edgell et al. [9] found that 39.6% of Americans think that atheists do not agree with their vision of American society and 47.6% would disapprove if their child married an atheist. Galen [11] reviewed several studies showing that Americans associate religiosity with moral behaviour. There is thus research showing that people in the USA consider religiosity to be favourable and lack of religiosity or atheism to be unfavourable [11].

Muslims are nearly as disliked as atheists in the USA and in contrast to atheists, Muslims are viewed as a mostly external threat, according to Edgell et al. [9]. According to Gerges [19], this started with the Islamic revolution in Iran. After the terrorist attacks on September 11, 2001, perceptions of Muslims also became more negative in the USA [20].

Sweden is a historically Christian country [21]. Many Swedes are members of the Church of Sweden and participate in rituals such as baptism and funerals, despite not believing in God. This identification with the religious past without religious beliefs is called cultural religion [22]. During the last decades, Sweden has received immigrants from more religious countries, both Christian and Muslim [23]. In 2021, 2,090,503 individuals in Sweden were born in another country [24]. This development might affect Swedes’ perceptions of religious people—especially Muslims, who have not historically been a large group in Sweden [21].

A European survey found that in Sweden, more people are opposed to having a Muslim neighbour than are opposed to having an immigrant neighbour [25]. Two Swedish studies found that a lost letter placed in a public place was less likely to be posted by a stranger if addressed to a Muslim name, rather than a common Swedish name [26]. A study conducted in Sweden and three other European countries found that participants were less willing to grant a Muslim immigrant citizenship than granting a Christian immigrant citizenship [27]. Thus, both in the USA and in Sweden, Muslims seem to be disliked. These studies did not differentiate between the attitudes of atheists and Christians toward Muslims.

The present studies contribute to the understanding of perceptions of atheists, Christians, and Muslims in Sweden, using participants from the USA as a benchmark, by exploring how people in these two countries rate atheist, Christian and Muslim job applicants on competence and likeability. Previous studies have investigated bias in recruitment pertaining to for example hairstyles [28], ethnicity [29], and gender [30]. There are also several studies which have found no bias [31] or negligible bias [32, 33], specifically when structured interviews are used. Eriksson et al. [34] investigated Swedish employers’ decisions to offer applicants a job based on a number of factors, including applicants’ religion. They found that Jewish and Muslim applicants were significantly less likely than Christian applicants to be offered a job. Drydakis [35] sent out real applications to job vacancies in Greece and varied whether the applicant indicated belonging to a religious minority (Pentecostal, evangelical or Jehovah’s Witness) or left out any information on religiosity (which would indicate that they belonged to the majority religion, Greek Orthodox Christianity). They found that religious minority applicants were less frequently invited to interviews and were offered lower wages.

The large number of previous studies that have investigated judgements of people based on group membership in recruitment contexts have mainly aimed to investigate bias and discrimination against these groups. The studies presented here could be interpreted from that perspective. However, our aim is to investigate perceptions of religious and nonreligious groups, especially whether these perceptions differ between in- and outgroups.

Study 1 was a pilot study intended to test the paradigm in a Swedish sample. In Study 2, the same experiment was conducted in a sample from the USA. In Study 3, Swedish participants saw one of three job descriptions with varying levels of responsibility, rated two applicants for the same job and subsequently chose one applicant who they would have hired. The hiring decision was added in order to increase statistical power and reduce possible social desirability bias by forcing participants to make a choice between the target (atheist, Christian or Muslim) and control (applicant who did not mention religion). Study 4 was identical to Study 3, but with participants from the USA.

## Study 1

### Method

The study described in Moss-Racusin et al. [30] was used as a template in the present studies. They investigated professors’ gender bias in recruitment, by asking them to rate an application for a position as laboratory manager and randomly assigning a male or female name. Participants were presented with minor demographic information, a cover letter, and an excerpt from a recommendation letter. They were then asked to rate the applicant on a number of items related to competence, hireability, salary, willingness to mentor, and likeability. Instead of varying the name of the applicant, the four studies presented here varied the religious affiliation of the applicant. Fewer demographic details were shown and the cover letter was replaced by shorter notes from an interview. Moreover, notes from a phone call with a reference person were shown instead of a recommendation letter excerpt. In addition, participants rated the applicants on seven instead of 21 items. The purpose of this was to minimise the time required for participants, and also to allow for several applicants to be evaluated. The expectation was that preferences for or against religious groups could be captured using this paradigm.

#### Participants

Participants were recruited from a participant pool, consisting mainly of students. They were not paid for participating in the study. Only people who reported living in Sweden, understanding written Swedish without problems and being at least 18 years old were able to participate. Of the 67 participants who completed the study, 7 were excluded for failing an attention check. The remaining 60 participants (34 female, 26 male, age *M* = 26.5, range 19–63), were included in analyses. Table 1 displays the gender, age, and religious affiliation of participants.

**Table 1:** Demographic data separated by participant religiosity in Study 1

	Atheist/none	Agnostic	Christian	Total
	(*N* = 32)	(*N* = 17)	(*N* = 10)	(*N* = 60)
Gender				
% Female	56.3	52.9	70	56.7
% Male	43.8	47.1	30	43.3
Age				
*M* (*SD*)	27.8 (8.6)	25.6 (6.9)	23.8 (2.7)	26.5 (7.4)
Education				
% Some HS	0	0	0	0
% Completed HS	3.1	11.8	30	11.7
% Some uni	50	52.9	40	48.3
% Bachelor	18.8	29.4	20	21.7
% Master	21.9	5.9	10	15
% Some doctoral	6.3	0	0	3.3
% PhD	0	0	0	0

Gender, age, and education of participants identifying as atheist/none, agnostic (agnostic, has not decided, or believe in higher powers but no organized religion) or Christian (all religious participants in this study were Christians). One participant chose “other” on the religious affiliation question and is included in the total column

*HS* high school, *uni* university

To investigate differences in ratings between participants of differing religious affiliations, they were categorised into three groups: atheists/nones, agnostics, and Christians. People who belonged to a religion other than Christianity were not included in these analyses. The first group included participants who identified as atheists or chose the option *none* when asked about their religious affiliation. There were several reasons that these participants were categorised together. First, especially in the USA (see study 2 and 4), the label *atheist* might have negative connotations for some people and they might therefore choose the more neutrally valenced *none* label. Second, there were a large number of available options for participants to choose from, including “has not decided”, “believe in higher powers, but no organised religion” and “agnostic”. Moreover, participants had the option to choose “other” and write their answer in their own words. Thus, the participants who felt that these options did not reflect their ideas of religion better than the option *none* are unlikely to be agnostics or believers who lack affiliation with an organised religious group. Third, there were a relatively large number of nones in studies 2 and 4, which together with atheists made up a large enough sample to make analyses with sufficient power. Fourth, mean ratings of nones and atheists for the different target groups followed the same patterns.

Agnostics were participants who had no clear target ingroup and were somewhere in between religious and atheistic. This group included participants who chose the option *believe in a higher power, but no organised religion*, *has not decided*, or *agnostic*. Participants categorised as Christians identified as *Christian (Catholic)*, *Christian (Baptist)* or *Christian (other)*. These categories were not planned before data collection. In the remainder of this paper, the terms *atheists*, *agnostics*, and *Christians* will refer to these groups, unless a different meaning is specified. Atheists, agnostics, and Christians differed in their strength of belief in God and, in most cases, in the role religion played in their lives (see Additional file 2).

#### Procedure

Participants completed the study online. After giving informed consent, participants were provided with general instructions, telling them that they would be asked to assume the role of a recruiter and rate fictitious applicants for different jobs. They were informed that they would receive notes from an interview done by another recruiter as well as a short description of the job. The two first cases were practice cases, constructed to give an example of a highly qualified applicant (for a position as cleaner) and a less qualified applicant (for a position as waiter). However, participants were not told that these cases were for practice. The two following cases were counterbalanced for gender of applicant, case order and experimental/control order. One of the cases concerned a position as a teacher for grades 1–6 and the other concerned a position as a personal care aide for a young child. The applicant in the teacher case was described as being active in a *Christian*, *Muslim*, *atheist,* or *interest* organisation—the last alternative acting as control. The applicant for the position as personal care aide was described as participating in a *Christian*, *Muslim*, *atheist,* or *philosophical* discussion group in her or his spare time—the last alternative acting as control. Each participant received both one experimental case (target: Christian, Muslim, or atheist) and one control case. For each case, participants answered seven 7-point Likert scale questions related to the competence and likeability of the applicant.

After rating the four applicants, participants completed an attention check, a question about their perceived aim of the study and several demographic questions. Finally, they were informed of the aim of the study. The instructions, job descriptions, information about participants, attention check, perceived aim of the study, and demographic and attitude measures can be found in Additional file 1.

#### Measures

Participants answered the following four questions related to the competence of the applicant: (1) When you read the qualities presented as important in the job description, how well-suited do you think the applicant is for the position? [very well-suited—not at all] (2) How competent does the applicant seem to be? [very competent—not at all] (3) How willing would you be to hire the applicant for the position? [very willing—not at all] (4) How probable do you think it is that the applicant receives the position? [very probable—not at all]. The next three questions concerned the likeability of the applicant: (5) How much do you think you would like the applicant? [very much—not at all] (6) Would you describe the applicant as someone you would like to get to know better outside of work? [very much—not at all] (7) Do you think that the applicant would fit in well with colleagues at the workplace? [very probable—not at all]. All text was in Swedish. A principal component analysis of the seven 7-point Likert scale questions about applicants’ competence and likeability in the first case made by participants in all four studies (*N* = 1374) found 2 components with an Eigenvalue greater than 1. The competence questions loaded on one factor while the likeability questions loaded on the other. The cumulative explained variance was 69.9%. The competence questions and the likeability questions were combined into two indexes.

## Results

No significant differences were found between the experimental conditions (atheist, Christian, or Muslim target) and the control condition on ratings of competence, *F*(1, 59) = 1.02, *p* = 0.317 or likeability, *F*(1, 59) = 1.42, *p* = 0.237. There were also no significant differences between targets in competence ratings, *F*(2, 57) = 0.41, *p* = 0.667 or likeability ratings, *F*(2, 57) = 0.49, *p* = 0.615. No interaction between participant religiosity (Christian, agnostic, or atheist) and target (atheist, Christian, or Muslim) was found for the competence score difference between the experimental case and the control case, *F*(4, 50) = 0.59, *p* = 0.670 or likeability score difference between experimental and control case, *F*(4, 50) = 2.31, *p* = 0.071. In Table 2, competence and likeability ratings for each participant religiosity group are shown.

**Table 2:** Competence and likeability ratings separated by participant religiosity in Study 1

	*N*	Competence				Likeability			
	Atheist/none, agnostic, Christian	Atheist/none	Agnostic	Christian	Total	Atheist/none	Agnostic	Christian	Total
		*M* (*SD*)	*M* (*SD*)	*M* (*SD*)	*M* (*SD*)	*M* (*SD*)	*M* (*SD*)	*M* (*SD*)	*M* (*SD*)
Atheist target	8, 6, 3	5.1 (0.9)	5.3 (0.6)	5.3 (0.4)	5.2 (0.7)	5.1 (1.1)	4.9 (0.7)	4.6 (0.7)	4.9 (0.9)
Control		4.8 (1.0)	4.9 (1.0)	5.6 (1.1)	5.0 (1.0)	4.9 (0.9)	5.3 (0.9)	4.2 (0.2)	4.9 (0.9)
Christian target	10, 6, 5	5.1 (0.9)	5.3 (0.7)	5.9 (1.1)	5.3 (0.9)	3.8 (1.0)	4.9 (0.8)	4.9 (0.8)	4.4 (1.0)
Control		4.7 (1.1)	4.8 (0.9)	5.9 (0.7)	5.0 (1.1)	4.6 (0.9)	4.8 (0.9)	4.6 (0.4)	4.7 (0.8)
Muslim target	14, 5, 2	5.3 (1.1)	4.9 (0.8)	5.0 (0.0)	5.2 (0.9)	4.5 (1.3)	4.7 (0.8)	5.5 (0.2)	4.6 (1.1)
Control		5.0 (0.7)	5.5 (0.6)	6.1 (0.5)	5.2 (0.7)	4.4 (1.0)	5.4 (0.6)	5.7 (0.9)	4.8 (1.0)
All targets	32, 17, 10	5.2 (0.9)	5.2 (0.7)	5.5 (0.8)	5.2 (0.8)	4.4 (1.3)	4.8 (0.7)	4.9 (0.7)	4.6 (1.0)
Control		4.9 (0.9)	5.1 (0.9)	5.8 (0.8)	5.1 (0.9)	4.6 (0.9)	5.2 (0.8)	4.7 (0.7)	4.8 (0.9)

Competence and likeability ratings for atheist, Christian and Muslim targets, all targets, and control ratings for participants identifying as atheist/none, agnostic (agnostic, has not decided, or believe in higher powers but no organized religion) or Christian (all religious participants in this study were Christians)

## Study 2

### Method

#### Participants

Participants were recruited through the site Prolific and were paid 1.25 GBP for participating. They had been pre-screened before recruitment for country of residence and political views. Only people who reported living in the USA were invited to participate. A third of the sample were recruited from people who had reported being politically liberal, another third from moderates and the remaining third from conservatives. This was done to get a more diverse sample, since the Prolific recruitment pool has a large overrepresentation of liberals. Of the 504 participants who completed the study, 48 were excluded (45 failed the attention check, 3 lived outside of the USA). Of the remaining 456 participants (225 female, 224 male, 7 other, age *M* = 37, range 18–79), who were included in analyses, 32.7% were conservative, 34.2% were moderate and 33.1% were liberal. Table 3 displays the gender, age, ideology, and religious affiliation of participants. The same participant religiosity categories as in study 1 were used.

**Table 3:** Demographic data separated by participant religiosity in Study 2

	Atheist/none	Agnostic	Christian	Total
	(*N* = 95)	(*N* = 108)	(*N* = 230)	(*N* = 456)
Gender				
% Female	43.2	50	52.2	49.3
% Male	55.8	47.2	47.4	49.1
% Other	1.1	2.8	0.4	1.5
Age				
M (SD)	34.2 (10.6)	35.1 (11.8)	40.0 (12.7)	37.4 (12.2)
Education				
% Some HS	0	1.9	1.7	1.3
% Completed HS	12.6	13	13	13.2
% Some uni	33.7	39.8	28.3	31.8
% Bachelor	37.9	27.8	37.8	35.5
% Master	11.6	16.7	16.5	15.6
% Some doctoral	1.1	0.9	0.9	0.9
% PhD	3.2	0	1.7	1.8
Ideology				
% Conservative	15.8	9.3	50.4	32.7
% Moderate	30.5	36.1	35.7	34.2
% Liberal	53.7	54.6	13.9	33.1

Gender, age, education, and ideology of participants identifying as atheist/none, agnostic (agnostic, has not decided, or believe in higher powers but no organized religion), or Christian. Muslim, Buddhist, and Jewish participants, as well as participants who chose the option “other” on the religious affiliation question, are included in the total column

*HS* high school, *uni* university

#### Procedure and measures

The procedure and measures were identical to Study 1, with the exceptions that all text was in English and that after being informed of the aim of the study, participants were provided with a link which allowed them to receive their payment for participating. This study was preregistered (https://osf.io/d4kg5).

### Results

When all targets were included, participants rated the applicant who revealed information about their religion (experimental conditions: atheist, Christian, or Muslim) as significantly less competent than the applicant who did not mention religion (control condition), *F*(1, 453) = 9.76, *p* = 0.002, and also rated the applicant as less likeable, *F*(1, 453) = 35.66, *p* < 0.001. There were no significant differences between targets (atheist, Christian or Muslim) in competence ratings, *F*(2, 453) = 1.20, *p* = 0.301 or likeability ratings, *F*(2, 453) = 2.60, *p* = 0.076.

**Fig. 1:**
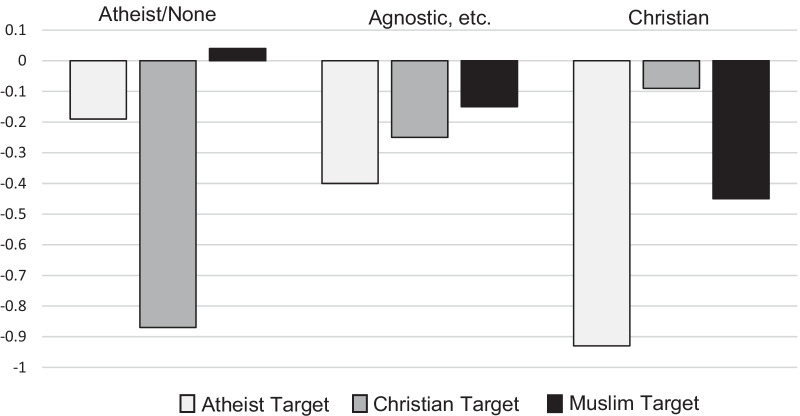
Likeability difference score between experimental case and control case separated by participant religiosity in Study 2. Likeability difference score between experimental case and control case for atheist, Christian and Muslim targets rated by participants identifying as atheist/none, agnostic (agnostic, has not decided, or believe in higher powers but no organized religion) or Christian

There was no interaction between participant religiosity (Christian, agnostic, or atheist) and target (atheist, Christian, or Muslim) on competence score difference between the experimental case and the control case, *F*(4, 436) = 1.40, *p* = 0.233. However, there was a significant interaction between participant religiosity and target on likeability score difference, *F*(4, 424) = 3.77, *p* = 0.005. Bonferroni post hoc tests showed that Christian participants rated the Christian target significantly higher than the atheist target (*p* = 0.002, *d* = 0.53). Moreover, compared to participants who were categorized as atheists or nones, Christian participants rated the Christian target significantly higher on likeability (*p* = 0.017, *d* = 0.57). No significant differences were found for the Muslim target. Adding ideology as a covariate did not change the results of the likeability analyses presented above. The analyses based on participant religiosity were not planned before data collection. In Table 4, competence and likeability ratings for each participant religiosity group are shown. Figure 1 presents the difference score between control and target ratings of likeability for each target and each participant religiosity group.

**Table 4:** Competence and likeability ratings separated by participant religiosity in Study 2

	*N*	Competence				Likeability			
	Atheist/none, agnostic, Christian	Atheist/none	Agnostic	Christian	Total	Atheist/none	Agnostic	Christian	Total
		*M* (*SD*)	*M* (*SD*)	*M* (*SD*)	*M* (*SD*)	*M* (*SD*)	*M* (*SD*)	*M* (*SD*)	*M* (*SD*)
Atheist target	33, 39, 78	4.8 (1.6)	4.6 (1.3)	4.5 (1.3)	4.6 (1.4)	4.9 (1.5)	4.7 (1.2)	4.2 (1.5)	4.5 (1.4)
Control		5.1 (1.1)	5.0 (1.2)	5.0 (1.0)	5.0 (1.1)	5.1 (1.2)	5.1 (1.0)	5.1 (1.0)	5.1 (1.0)
Christian target	36, 29, 74	4.5 (1.3)	4.8 (1.2)	4.9 (1.2)	4.7 (1.2)	4.0 (1.3)	4.6 (0.8)	4.9 (1.2)	4.6 (1.2)
Control		5.0 (1.2)	4.9 (1.0)	5.0 (1.3)	5.0 (1.2)	4.8 (0.9)	4.8 (1.0)	5.0 (1.3)	4.9 (1.1)
Muslim target	26, 40, 78	5.0 (1.3)	5.2 (0.8)	4.5 (1.4)	4.8 (1.3)	4.9 (1.3)	5.1 (0.9)	4.5 (1.2)	4.8 (1.2)
Control		4.5 (1.3)	5.1 (1.1)	4.9 (1.2)	4.9 (1.2)	4.8 (1.3)	5.2 (0.9)	4.9 (1.1)	5.0 (1.1)
All targets	95, 108, 230	4.7 (1.4)	4.9 (1.1)	4.7 (1.3)	4.7 (1.3)	4.5 (1.4)	4.8 (1.0)	4.6 (1.3)	4.6 (1.3)
Control		4.9 (1.2)	5.0 (1.1)	5.0 (1.2)	5.0 (1.2)	4.9 (1.1)	5.1 (1.0)	5.0 (1.1)	5.0 (1.1)

Competence and likeability ratings for atheist, Christian, and Muslim targets, all targets, and control ratings for participants identifying as atheist/none, agnostic (agnostic, has not decided, or believe in higher powers but no organized religion), or Christian. Participants who identified as Muslim, Buddhist, or Jewish, as well as participants who chose the option “other” on the religious affiliation question, are included in the total column

## Study 3

### Method

#### Participants

Of the 449 participants who completed the study, 135 were recruited from social media sites (Facebook and Reddit). They were not paid for participating in the study. The remaining 314 participants were recruited from Prolific and were paid 1.00 GBP for their participation. Only people who reported living in Sweden, understanding written Swedish without problems, and being at least 18 years old were invited to participate. Of the 449 participants who completed the study, 58 were excluded for failing the attention check and 2 were excluded due to reporting that they did not understand written Swedish without problems. The remaining 389 participants (271 female, 112 male, 6 other, age *M* = 29.84, range 18–95), were included in analyses. Table 5 displays gender, age, and religious affiliation of the participants. The same participant religiosity categories as in study 1 and 2 were used.

**Table 5:** Demographic data separated by participant religiosity in Study 3

	Atheist/none	Agnostic	Christian	Total
	(*N* = 85)	(*N* = 108)	(*N* = 246)	(*N* = 470)
Gender				
% Female	55.3	46.3	54.9	52.6
% Male	41.2	51.9	44.7	46.2
% Other	3.5	1.9	0.4	1.3
Age				
*M* (*SD*)	32.71 (11.0)	29.1 (9.6)	30.4 (9.6)	30.7 (10.2)
Education				
% Some HS	3.5	0	0.4	0.9
% Completed HS	10.6	17.6	10.2	11.9
% Some uni	27.1	36.1	24.8	27.7
% Bachelor	40	32.4	45.5	41.1
% Master	12.9	11.1	16.7	15.5
% Some doctoral	1.2	0	0.4	0.4
% PhD	4.7	2.8	2	2.6
Ideology				
% Conservative	17.6	19.4	47.2	33.4
% Moderate	21.2	30.6	39.4	33
% Liberal	61.2	50	13.4	33.6

#### Procedure

The procedure was similar to the previous two studies. However, participants rated two applicants for the position as a cleaner (as practice cases) and then two applicants applying for another job, one at a time. This second job was a position as a personal care aide, a teacher, or an administrative director of a municipality’s unit for economic support for people in need. This would approximate a regional commissioner of the Social Security Administration in the USA, which was used in the English translation used in study 4. It was implied that one of the two latter applicants was an atheist, a Christian, or a Muslim (through the same sentences as used in study 1), while no information about religiosity was revealed about the other applicant. After rating the two applicants in the practice cases and the two applicants applying for one of the aforementioned positions, using the same questions as in Study 1, applicants were asked to choose one of the two latter applicants to hire for the job. The remainder of the study was identical to Study 1. This study was preregistered (https://osf.io/zhbu4/).

#### Measures

In addition to the seven questions used in the previous studies, participants chose one of the two last participants to hire.

### Results

Similarly to Study 2, a significant difference in likeability, *F*(1, 388) = 5.86, *p* = 0.016 was found between targets and control cases, meaning that participants rated applicants who did not mention religion higher than applicants who were atheists, Christians, or Muslims. No significant difference in competence between targets and control was found, *F*(1, 388) = 3.11, *p* = 0.079.

**Fig. 2:**
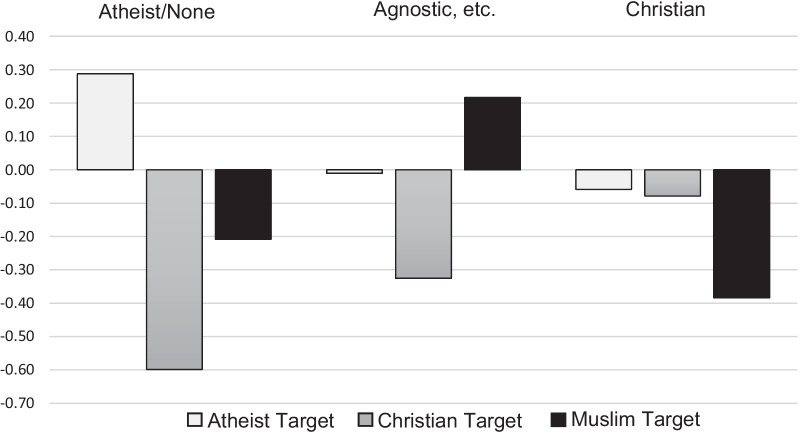
Likeability difference score between experimental case and control case separated by participant religiosity in Study 3. *Note* Likeability difference score between experimental case and control case for atheist, Christian, and Muslim targets rated by participants identifying as atheist/none, agnostic (agnostic, has not decided, or believe in higher powers but no organized religion), or Christian

No significant difference between target groups in competence ratings was found, *F*(2, 386) = 0.93, *p* = 0.396, but the difference between targets in likeability ratings was significant, *F*(2, 386) = 7.80, *p* < 0.001. Bonferroni post hoc tests showed that the Christian target was rated as less likeable than both the atheist target (*p* < 0.001) and the Muslim target (*p* = 0.025). The interaction between participant religiosity and target in likeability ratings was not significant, *F*(4, 354) = 2.24, *p* = 0.064, but the interaction for competence ratings was statistically significant, *F*(4, 354) = 3.09, *p* = 0.016. Bonferroni post hoc tests showed that Christian participants rated the Christian target higher in competence than the Muslim target (*p* = 0.045, *d* = 0.77). Since the interaction for likeability ratings was close to being statistically significant, Bonferroni post-hoc tests were done to further investigate possible differences between participant religiosity groups. These found that atheist participants rated atheist targets as being more likeable than both Christian targets (*p* < 0.001, *d* = 0.87) and Muslim targets (*p* = 0.026, *d* = 0.47). No other likeability ratings differed significantly. In Table 6, competence and likeability ratings for each participant religiosity group are shown. Figure 2 presents the difference score between control and target ratings of likeability for each target and each participant religiosity group.

**Table 6:** Competence and likeability ratings separated by participant religiosity in Study 3

	*N*	Competence				Likeability			
	Atheist/none, agnostic, Christian	Atheist/none	Agnostic	Christian	Total	Atheist/none	Agnostic	Christian	Total
		*M* (*SD*)	*M* (*SD*)	*M* (*SD*)	*M* (*SD*)	*M* (*SD*)	*M* (*SD*)	*M* (*SD*)	*M* (*SD*)
Atheist target	66, 33, 17	5.0 (1.3)	5.0 (1.1)	4.9 (1.0)	5.0 (1.2)	4.8 (1.1)	4.7 (1.2)	4.7 (0.9)	4.8 (1.1)
Control		4.9 (1.1)	5.1 (1.3)	5.1 (1.0)	5.0 (1.2)	4.6 (1.3)	4.7 (1.1)	4.8 (0.9)	4.7 (1.2)
Christian target	59, 39, 17	5.2 (0.9)	5.0 (0.9)	5.25 (1.09)	5.1 (1.0)	4.3 (1.0)	4.5 (1.0)	4.8 (1.1)	4.4 (1.1)
Control		5.5 (1.0)	5.1 (0.9)	5.0 (1.3)	5.3 (1.0)	4.9 (1.0)	4.8 (1.0)	4.9 (1.2)	4.9 (1.0)
Muslim target	72, 40, 20	5.3 (1.1)	5.1 (1.2)	4.9 (1.1)	5.2 (1.1)	4.7 (1.2)	5.0 (1.1)	4.3 (1.7)	4.8 (1.3)
Control		5.4 (1.0)	5.0 (1.3)	5.4 (0.9)	5.2 (1.1)	4.9 (1.1)	4.8 (1.2)	4.7 (0.9)	4.8 (1.1)
All targets	197, 112, 54	5.2 (1.1)	5.0 (1.1)	5.0 (1.1)	5.1 (1.1)	4.6 (1.1)	4.7 (1.1)	4.6 (1.3)	4.7 (1.2)
Control		5.3 (1.1)	5.1 (1.2)	5.2 (1.1)	5.2 (1.1)	4.8 (1.1)	4.8 (1.1)	4.8 (1.0)	4.8 (1.1)

Competence and likeability ratings for atheist, Christian, and Muslim targets, all targets, and control ratings for participants identifying as atheist/none, agnostic (agnostic, has not decided, or believe in higher powers but no organized religion), or Christian. Muslim, Buddhist, Hindu, and Jewish participants, as well as participants who chose the option “other” on the religious affiliation question, are included in the total column

A chi-square test was used to examine if there was an association between hiring atheists, Muslims, and Christians, rather than hiring control applicants, who did not mention religion. This showed a significant association χ^2^(2) = 13.31, *p* = 0.001. Atheist targets (62 participants, 50.4%), Christian targets (43 participants, 33.9%) and Muslim targets (77 participants, 55.4%) were hired to different extents. Overall, 182 participants hired the target applicant, while 207 participants hired the control applicant. However, when the test was done for each participant religiosity group (atheists/none, agnostics, and Christian) separately, the association was only statistically significant for atheists’ hiring decisions of atheist targets (35 participants, 53.0%), Christian targets (14 participants, 23.7%), and Muslim targets (36 participants, 50.0%), χ^2^(2) = 13.08, *p* = 0.001, but not for agnostics’ hiring decisions of atheist targets (17 participants, 51.5%), Christian targets (16 participants, 41.0%), and Muslim targets (25 participants, 62.5%), χ^2^(2) = 3.65, *p* = 0.161 and Christian participants’ hiring decisions of atheist targets (7 participants, 53.0%), Christian targets (10 participants, 58.8%), and Muslim targets (10 participants, 50.0%), χ^2^(2) = 1.06, *p* = 0.589. Atheists hired Christian targets significantly less frequently than applicants who did not mention their religion (*p* = 0.0003), but no other associations regarding hiring decisions were statistically significant. This p-value was obtained by using the adjusted residuals from the chi-square test, multiply these values with themselves and using the Sig.Chisq function in SPSS (compute variable) with 1 df to obtain p-values. These were then compared to a corrected p-value (p = 0.0028, since 18 tests were made) and interpreted as significant if they fell below this corrected p-value. This method of post-hoc testing chi-square analyses is described in García-Pérez et al. [36].

## Study 4

### Method

#### Participants

Participants were recruited through Prolific and were paid 1.25 GBP for participating. They had been pre-screened before recruitment for nationality and political views. Only people who reported being US citizens were invited to participate. As in Study 2, a third of the sample were recruited from people who had reported being politically liberal, another third from moderates, and the remaining third from conservatives. Of the 504 participants who completed the study, 34 were excluded (30 failed the attention check, 4 lived outside of the USA). Of the remaining 470 participants (247 female, 217 male, 6 other, age *M* = 31, range 18–73), who were included in analyses, 33.4% were conservative, 33.0% were moderate, and 33.6% were liberal. Table 7 displays the gender, age, ideology, and religious affiliation of participants. The same participant religiosity categories as in study 1, 2 and 3 were used.

**Table 7:** Demographic data separated by participant religiosity in Study 4

	Atheist/None	Agnostic	Christian	Total
	(*N* = 85)	(*N* = 108)	(*N* = 246)	(*N* = 470)
Gender				
% Female	55.3	46.3	54.9	52.6
% Male	41.2	51.9	44.7	46.2
% Other	3.5	1.9	0.4	1.3
Age				
*M* (*SD*)	32.71 (11.0)	29.1 (9.6)	30.4 (9.6)	30.7 (10.2)
Education				
% Some HS	3.5	0	0.4	0.9
% Completed HS	10.6	17.6	10.2	11.9
% Some uni	27.1	36.1	24.8	27.7
% Bachelor	40	32.4	45.5	41.1
% Master	12.9	11.1	16.7	15.5
% Some doctoral	1.2	0	0.4	0.4
% PhD	4.7	2.8	2	2.6
Ideology				
% Conservative	17.6	19.4	47.2	33.4
% Moderate	21.2	30.6	39.4	33
% Liberal	61.2	50	13.4	33.6

Gender and age of participants identifying as atheist/none, agnostic (agnostic, has not decided, or believe in higher powers but no organized religion), or Christian. Hindu, Buddhist, Muslim, Jewish, and Sikh participants (*N* = 24), as well as participants who chose “other” on the religious affiliation question (*N* = 7), are included in the total column

*HS* high school,* uni* university

#### Procedure and measures

The procedure and measures were identical to Study 3, with the exception that all text was in English. This study was preregistered (https://osf.io/zhbu4/).

#### Hypotheses

Several hypotheses were specified in the preregistration. As found in study 2, it was hypothesised that (1) when information about applicants’ religion is available, they will be rated as (a) less competent and (b) less likable than when such information is not available. As differences in likeability ratings of the different targets based on participant religiosity had been found in study 2, it was hypothesised that (2) religious participants would rate atheist applicants as less likeable than Christian applicants and (3) atheist applicants would rate Christian applicants as less likeable than atheist applicants. Regarding hiring decisions, it was hypothesised that (4) religious participants would hire Christian applicants more often than atheist applicants and (5) atheist participants would hire atheist applicants more often than Christian applicants. It was subsequently decided that all non-Christians in the religious group would be excluded from the analyses grouping participants based on religiosity. Thus, the group *religious participants* mentioned in hypotheses (2) and (4) in the preregistration were changed to *Christian participants* after the preregistration was published.

### Results

When all targets were included, participants rated the applicant who revealed information about their religion (experimental conditions: atheist, Christian, or Muslim) significantly less competent than the applicant who did not mention religion (control condition), *F*(2, 467) = 4.46, *p* = 0.012, and also as less likeable, *F*(2, 467) = 6.47, *p* = 0.002. Thus, hypotheses (1a) and (1b) were supported. Significant differences between targets in competence ratings, *F*(2, 467) = 4.46, *p* = 0.012 and likeability ratings, *F*(2, 467) = 6.47, *p* = 0.002 were also found. Bonferroni post hoc tests showed that the Muslim target was rated as more competent (*p* = 0.016, *d* = 0.31) and more likeable (*p* = 0.001, *d* = 0.40) than the atheist target.

No interaction was found between participant religiosity (Christian, agnostic, or atheist) and target (atheist, Christian, or Muslim) on competence score difference between the experimental case and the control case, *F*(4, 430) = 1.04, *p* = 0.386. Similarly to Study 2, an interaction was found between participant religiosity and target on likeability score difference, *F*(4, 430) = 5.18, *p* < 0.001. Bonferroni post-hoc tests showed that Christian participants rated atheist targets as less likeable than both Christian (*p* = 0.001, *d* = 0.53) and Muslim targets (*p* = 0.001, *d* = 0.03), in comparison with the control ratings. Thus, hypothesis (2) was supported. Atheists rated Christian targets as less likeable than both atheist targets (p = 0.015, *d* = 0.75) and Muslim targets (*p* = 0.015, *d* = 0.79), in comparison with control ratings. This means that hypothesis (3) was supported as well. Atheist targets were rated as more likeable by atheist participants than by Christian participants (*p* = 0.006, *d* = 0.65) and Christian targets were rated as more likeable by Christian participants than by atheist participants (*p* = 0.011, *d* = 0.63). Adding ideology as a covariate did not change the results of the likeability analyses presented above.

**Fig. 3:**
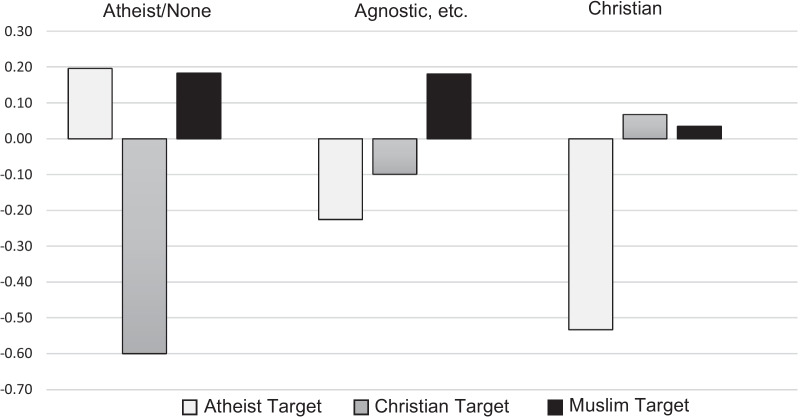
Likeability difference score between experimental case and control case separated by participant religiosity in Study 4. *Note* Likeability difference score between experimental case and control case for atheist, Christian, and Muslim targets rated by participants identifying as atheist/none, agnostic (agnostic, has not decided, or believe in higher powers but no organized religion) or religious (Christian, Hindu, Buddhist, Jewish or Sikh)

In Table 8, competence and likeability ratings for each participant religiosity group are shown. Figure 3 presents the difference score between control and target ratings of likeability for each target and each participant religiosity group.

**Table 8:** Competence and likeability ratings separated by participant religiosity in Study 4

	*N*	Competence				Likeability			
	Atheist/none, agnostic, Christian	Atheist/none	Agnostic	Christian	Total	Atheist/none	Agnostic	Christian	Total
		*M* (*SD*)	*M* (*SD*)	*M* (*SD*)	*M* (*SD*)	*M* (*SD*)	*M* (*SD*)	*M* (*SD*)	*M* (*SD*)
Atheist target	29, 34, 80	4.9 (1.1)	5.0 (1.2)	4.9 (1.2)	4.9 (1.2)	4.9 (1.1)	5.0 (1.1)	4.7 (1.4)	4.8 (1.3)
Control		4.8 (1.1)	5.3 (1.1)	5.2 (1.1)	5.1 (1.1)	4.7 (1.1)	5.2 (1.1)	5.2 (1.1)	5.1 (1.1)
Christian target	25, 37, 79	5.0 (1.1)	5.3 (1.4)	5.0 (1.2)	5.1 (1.3)	4.3 (1.3)	5.0 (1.2)	5.0 (1.2)	4.9 (1.2)
Control		5.0 (1.1)	5.3 (1.1)	4.9 (1.2)	5.0 (1.2)	4.9 (1.2)	5.1 (1.2)	4.9 (1.2)	5.0 (1.2)
Muslim target	31, 37, 87	4.7 (1.3)	4.9 (1.2)	4.9 (1.1)	4.9 (1.3)	4.8 (1.5)	4.8 (1.2)	4.8 (1.1)	4.9 (1.2)
Control		4.8 (1.3)	4.7 (1.4)	4.9 (1.2)	4.8 (1.3)	4.7 (1.5)	4.7 (1.5)	4.8 (1.2)	4.8 (1.4)
All targets	85, 108, 246	4.9 (1.2)	5.0 (1.3)	4.9 (1.2)	4.9 (1.2)	4.7 (1.3)	4.9 (1.1)	4.8 (1.2)	4.8 (1.2)
Control		4.9 (1.2)	5.1 (1.2)	5.0 (1.2)	5.0 (1.2)	4.7 (1.3)	5.0 (1.3)	5.0 (1.2)	4.9 (1.2)

Competence and likeability ratings for atheist, Christian, and Muslim targets, all targets, and control ratings for participants identifying as atheist/none, agnostic (agnostic, has not decided, or believe in higher powers but no organized religion), or Christian

To test whether there was an association between target (Christians, Muslims, and atheists) and participants’ decisions to hire the target rather than the control applicant, a chi-square test was used. A significant association was found χ^2^(2) = 6.23, *p* = 0.044, meaning that participants hired Christians (60 participants, 39.0%), Muslims (88 participants, 52.7%) and atheists (66 participants, 44.3%) to different extents. Overall, 214 participants (45.5%) hired the target, while 256 participants (54.5%) hired the control applicant, who did not mention religion. When the same analysis was done for each of the participant religiosity groups, there was no significant association between hiring decisions of Christian targets (7 participants, 28.0%), Muslim targets (14 participants, 45.2%), and atheist targets (17 participants, 58.6%) made by atheists/nones χ^2^(2) = 5.10, *p* = 0.074. Similarly, there were no significant association between hiring decisions made by agnostics for Christian targets (16 participants, 43.2%), Muslim targets (25 participants, 67.6%), and atheist targets (17 participants, 50.0%) χ^2^(2) = 4.68, *p* = 0.097. Finally, no association was found regarding hiring decisions of Christian targets (34 participants, 43.0%), Muslim targets (42 participants, 48.3%), and atheist targets (29 participants, 36.3%) made by Christian participants χ^2^(2) = 2.47, *p* = 0.291. Thus, hypotheses (4) and (5) were not supported.

## Discussion

In this paper four studies investigating Christian, Muslim, and atheist job applicants’ perceived competence and likeability were reported. Significant differences in likeability ratings were found in several studies, where atheists and Christians preferred the target belonging to their ingroup. Significant differences between groups in competence ratings or hiring decisions were rare. In the first pilot study, no statistically significant findings were observed. However, the sample consisted of only 60 participants, which likely was too few for possible differences to be detected. In the three subsequent studies with sufficient sample sizes, participants rated applicants who mentioned religion (or atheism) as being less likable than applicants for which religion was not mentioned. These results were found both in the USA and in Sweden. Although a significant difference was not reached in the Swedish sample, applicants who mentioned religion were rated as less competent in both studies with American participants. In study 2, conducted in a US sample, Christian participants rated the Christian target as being more likable than the atheist target and also rated the Christian target as more likeable than atheist participants did. These results were replicated in study 4, which also found that atheist participants rated Christian targets as being less likeable than both atheist targets and Muslim targets. Atheist targets were also rated as more likeable by atheist participants than by Christian participants. In study 3, with a Swedish sample, atheist participants rated atheist targets as more likable than both of the religious targets. They also hired applicants who did not mention religion (76.3%) more often than the Christian applicants (23.7%). However, Christian participants did not rate atheist targets as less likeable than Christian targets.

Mentions of religiosity or lack thereof led to lower likeability ratings in both Swedish and US samples, as well as lower competence ratings in the US samples. This might be explained by the information in the control conditions, where applicants reported being part of a philosophical discussion group or an interest organisation, being perceived as more favourable than being part of a religious/atheistic discussion group or organisation. It might also be explained by mentions of religious (dis)belief being more likely to be perceived as negative in a recruitment context by people in general.

When a difference in ratings between targets was found, it was consistently people preferring their religious ingroup over a religious outgroup. Significant results were however only found for likeability ratings and not in competence ratings, i.e., people did not view religious ingroup members as more competent, but merely as more likeable. The exception was in study 3, where Muslim targets were deemed less competent than Christian targets by Christian participants. In study 4, the differences in likeability ratings did not affect hiring decisions. However, in study 3 atheist participants, who rated atheist targets as more likeable but not more competent than Christian targets, hired Christians (23.7%) less often than applicants who did not mention religion (76.3%). Similar differences in hiring decision were not found for the other targets. Thus, in some cases likeability might affect hiring decisions, even when there is no significant difference in perceived competence.

Christian Swedes rated Muslim targets as less competent than Christian targets. This result is surprising, since it was the only significant difference in competence ratings in all included studies and since it was not coupled with significantly lower likeability ratings or lower willingness to hire Muslims. One possibility is that Christians in Sweden do not dislike Muslims more than they dislike atheists or other Christians but still view Muslims as less competent. Another possibility is that Christian Swedes are more comfortable with rating Muslims as less competent than to rate them as less likeable. Swedish Christians might consider it a moral obligation to love and value all people equally, despite religious differences. However, their religion does not encourage them to view everyone as equally competent. It is also possible that increased power would be needed in order to determine if they also view Muslim targets as less likeable.

The only significant difference in hiring decisions was that Swedish atheists hired Christian applicants less often than they hired control applicants. Surprisingly, no such effect was found in the USA. The Swedish atheists hired Christian targets in 23.7% of cases, while American atheists hired Christian targets in 28.0% of cases. It is possible that no significant association was found in the latter case due to the lower number of atheist participants in study 4. However, it is also possible that Swedish atheists are less willing to hire Christian applicants than American atheists are. In any case, the result from study 3 indicates that Christian applicants are less likely to be hired by atheist Swedes, which could imply that this group has a negative enough view of Christians to warrant avoiding hiring them.

When likeability ratings and hiring decisions differed between targets, religious ingroup members were preferred. This is in line with ample research showing that ingroup members are generally perceived to have more positive qualities. In study 3, Christians rated Christian targets as more competent than Muslim targets. Apart from this finding, an ingroup preference was not seen in any competence ratings, indicating that religious ingroup members are generally not viewed as more competent than outgroup members. It is possible that significant, albeit small, differences in competence ratings between targets could have been detected with larger samples.

In study 3, only atheist participants were found to rate their religious ingroup higher on likeability. This might be explained by the fact that they constituted the majority of the sample (110 atheists and 87 nones) and thus generated enough statistical power to produce a significant difference, while the 54 Christian participants were too few for an ingroup likeability preference to be detected. Speaking against this interpretation is the fact that the difference between Christians’ likeability ratings of atheist targets and Christian targets was very small. Another possibility is that the stigma against atheists, which has been demonstrated in the USA and supported by the results from study 2 and 4, is not that pervasive in Sweden. Being nonreligious is common and perhaps therefore not as penalised by Christians in Sweden.

Atheists and nones in study 4 rated Christian targets as less likeable than both the other targets, which means that they liked atheist applicants more than Christian applicants despite living in a country where numerous studies have found that the population prefer religious people over nonreligious people. Thus, this study indicates that ingroup favouritism influences likeability judgements made by atheists and nones in the USA more than the general stigma against atheists in the country. Alternatively, outgroup derogation directed at Christians might affect these ratings more than ingroup favouritism.

Previous studies have found little or no gender bias in recruitment contexts when structured interviews or structured employment references are used [32, 33, 37]. The lack of variation in competence ratings and hiring decisions across targets in the studies presented here might be due to the structured information that participants received. The order and amount of information about experience, education, personality, skills, and references was similar in all cases. If participants had evaluated a less structured motivation letter, the results might have been different. The results at least indicate that mentioning religious affiliation in a recruitment context might make an applicant seem both less likeable and less competent. Moreover, the differences in results between likeability and competence ratings indicate that religious ingroup favouritism or outgroup derogation seem to mainly affect likeability ratings and more seldom competence ratings.

### Limitations

The religious affiliation of targets was not directly stated, but rather implied by their involvement with an atheist, a Christian, or a Muslim group. This was necessary to avoid suspicion by participants, but means that some participants might have interpreted the information in an unintended way. In addition, participants might think that mentions of religiosity are improper in a recruitment context, which might be an explanation for the finding that applications that did not mention religion were rated higher. Another consequence of this design is that participants might perceive people who are actively involved in a discussion group or an organisation with a (non)religious focus as more extreme than people who simply identify with a religion or as atheists.

Even though Muslims are a minority group in both Sweden and the USA, with a culture that is relatively distant from both atheists and Christians, Muslims were rated as likeable and competent as atheists’ and Christians’ ingroups in most studies. The two exceptions were found in Study 3, where atheists rated Muslims as less likeable than atheist targets and Christians rated Muslims as less competent than Christian targets. This could mean that Muslims are well-liked in both countries, but a more probable interpretation would be social desirability bias. Participants might want to appear to be unprejudiced and thus rate Muslim targets higher than they otherwise would have done. The religious information when the applicant was a Muslim might have become more salient for participants than when the applicant was an atheist or a Christian, since being Muslim is more uncommon in the two countries where the studies were done.

There are a few discrepancies from the preregistration for study 2. The initial plan was to conduct another study in Sweden, identical to study 2. For practical reasons, we could not conduct two studies of that sample size in Sweden and opted for two new studies with a changed design (study 3 and 4) instead. Thus, the hypothesis and intended analyses that we preregistered could not be investigated. We also opted to use ANOVAs with difference scores for our analyses instead of repeated measures, as we had preregistered in study 2.

Since participants were aware that the applicants were fictitious, they might not respond as they would if they were asked about real people and real positions. A study where participants were asked about ostensibly real cases, similar to Moss-Racusin et al. [30], would avoid this limitation.

A limited number of jobs was used in the studies. Participants rated applicants’ likeability and competence in relation to the positions they applied for, meaning that religious affiliation might affect perceived competence or likeability differently had other types of jobs been used. The intention was to use positions which would require a high degree of moral responsibility—taking care of a defenceless person (personal care aide), teaching children (teacher), and making decisions affecting people’s financial situation (administrative director). Other jobs might be more heavily dependent on qualities such as intelligence, industriousness, or cooperativeness.

The USA is a large country with religiosity levels and other cultural aspects differing between states. Since participants anywhere in the USA could participate through Prolific, the results might not be generalisable to all parts of the country.

### Future research

Future studies could investigate jobs that require specific qualities, such as morality, industriousness, intelligence, and cooperativeness. Such studies could examine if people perceive atheists, Christians, or Muslims to have some of these qualities to a greater extent, if people perceive their religious ingroup members to possess these qualities to a greater degree, and if such perceptions differ between cultures, e.g., depending on the majority religion or religious history. In the present studies, jobs varying in responsibility and status were used. Future studies could investigate whether jobs of low or high status lead to different competence and likeability ratings for atheists, Christians, and Muslims, depending on the religious affiliation of participants. Another approach would be to ask participants to imagine themselves as being a customer or equivalent tasked with rating an employee, where similar information is available and religious belief is varied between conditions. This would make it possible to examine participants’ perceptions when they have more of a stake in the situation.

In conclusion, the present studies suggest that in some cases people in Sweden, as well as people in the USA, rate religious ingroup members as more likeable, but not more competent, than religious outgroup members.

## Supplementary Information

**Additional file 1.** Participant instructions, job descriptions, applicant information, attention check, demographic and attitude items.

**Additional file 2.** Additional analyses.

## Data Availability

The datasets generated and analysed during the current study are available from the OSF database (https://osf.io/htpu2/?view_only=bc80502157cf4371ac86aa85af2d660e).
