# Supplementary material for: Religious affiliation seldom seems to influence hiring or competence ratings of job applicants: studies conducted in Sweden and in the USA
Source: BMC Psychol. 2022 Sep 19;10:220. doi: 10.1186/s40359-022-00927-0 (PMC9484152; doi:10.1186/s40359-022-00927-0)
Supplement: Supplementary file 1 — Additional file 1. Participant instructions, job descriptions, applicant information, attention check, demographic and attitude items. [file 40359_2022_927_MOESM1_ESM.docx]

Material

In studies 1 and 3, all text was written in Swedish. Other than that, there were no differences between studies 1 and 2 or between studies 3 and 4, unless specified in the text below.

**Instructions**

In all four studies, participants received the following instructions.

*In this study, you will adopt the role of a recruiter and evaluate people who are applying for different types of jobs. You will first read a description of the job, where you can see which qualities are important. Then you will read notes from another recruiter, who has interviewed an applicant and thereafter we will ask you to rate how well the person fits the job.*

*The description of the job is meant to give you an idea of what the employer is requesting. In the interview notes things like gender, age, education and previous work experience is included. It also includes a summary of the most important outcomes of the interview (“other info from the interview”) as well as comments from the recruiter’s phone call with a reference given by the applicant.*

*Explanation of words:*

*Recruiter = person whose job it is to hire people.*

*Applicant = the person who has applied for a job.*

*Reference = a person who can give information about how the applicant is in his/her work role, such as a previous boss, colleague or supervisor/teacher from school.*

In study 3 and 4, the following sentence was also included.

*The same position can occur several times, with different applicants.*

**Job descriptions and applicant information**

**Studies 1 and 2**

***Practice Case: Cleaner***

Participants read the following job description.

*Cleaner*

*This job requires experience with cleaning work. The person should autonomously clean offices in the building two days a week, which is expected to take 16 hours altogether. Important personal qualities for the job are social competence and consideration, since the cleaner will work while other people are working in the offices.*

Participants then received the following information about the applicant.

*Gender: Woman*

*Age: 37*

*Previous work experience: Has worked as a cleaner for about 17 years, and spent a couple of years with administrative work.*

*Education: Courses in cleaning at Helge’s Cleaning Inc. and Cleaning Service Inc., equivalent to 10 weeks.*

*Other info from the interview: The applicant gives a very confident impression. Seems committed and interested in the position. Judging from her answers on the interview questions, as well as the general impression during the interview, the applicant seems to function very well socially.*

*Reference: The reference is a manager at Cleaning Service Inc. who reports that he is very pleased with the applicant’s work. The applicant has worked in offices before and is used to being considerate to people working while she is cleaning.*

After having read this information, participants rated the applicant on the seven 7-point Likert scale items regarding competence and likeability.

***Practice Case: Waiter or Waitress***

Participants read the following job description.

*Waiter or waitress*

*A stressful profession which requires service mindedness and an ability to work at a high pace. The work is mainly scheduled for evenings and weekends, and the person should be able to step in on short notice when more people have booked tables at the restaurant than expected. Experience of similar work is highly desirable. In order to make sure the teamwork between cooks, other waiters/waitresses and other staff works smoothly, the person should have good cooperation skills.*

Participants then received the following information about the applicant.

*Gender: Man*

*Age: 30*

*Previous work experience: Work as a nursing assistant in a geriatric care home, 2 years. Summer job at a rural café, 1 summer. He has a certificate of employment from the position as nursing assistant.*

*Education: High school diploma with specialization in vehicles.*

*Other info from the interview: Seems nervous and takes a long time to answer some questions. The level of energy is quite low, but this could be caused by the nervousness. He is unsure of whether he would be able to step in on short notice at the restaurant when needed.*

*Reference: The reference is a manager at the geriatric care home that the person worked at, but cannot say much more than that the person has been showing up on time and that she can’t remember hearing any complaints from colleagues*.

After having read this information, participants rated the applicant on the seven 7-point Likert scale items regarding competence and likeability.

***Teacher***

This case was always presented as case 3 or case 4 (counterbalanced). Participants were either told about the applicant’s religious affiliation (target: Christian, Muslim or atheist) or not (control). This was also counterbalanced, as was gender of the applicant. Participants read the following job description.

*Teacher grades 1-6*

*This position for a teacher at an elementary school entails that the person teaches one class from first to sixth grade in most school subjects. The person being hired should be a certified teacher. It is also important to have experience working with children and being able to handle children in the typical age span (6-12 years old) in a good way. This implies, for example, being able to stimulate the pupils’ appetite for learning, identify and act in accordance with each pupil’s individual needs as well as solving conflicts with pupils and between pupils. The position includes work with digital tools*.

Participants then received the following information about the applicant. In study 1, participants were told the applicant had their teaching degree from Högskolan Dalarna.

*Gender: [Woman/Man]*

*Age: 34*

*Previous work experience: Has worked as a teacher in a primary school (grades 1-3) for four years. Before college, [she/he] worked as a storekeeper.*

*Education: Certified teacher for kindergarten and grades 1-3 (Michigan State University). Has also complemented [her/his] education with courses to gain qualification for teaching grades 4-6, but has some courses left to take.*

*Other info from the interview: The applicant is well-spoken and seems to be a social and observant person. Says that [she/he] in [her/his] spare time is active in a [Christian/Muslim/atheist/interest] organization.*

*Reference: The reference is a teaching colleague at the applicant’s current workplace. [He/She] says that the applicant is a good teacher who is able to create a good atmosphere in the classroom. However, [he/she] mentions that teaching older children in grades 4-6 might be a challenge for [her/him].*

After having read this information, participants rated the applicant on the seven 7-point Likert scale items regarding competence and likeability.

***Personal Care Aide***

This case was always presented as case 3 or case 4 (counterbalanced). Participants were either told about the applicant’s religious affiliation (target: Christian, Muslim or atheist) or not (control). This was also counterbalanced, as was gender of the applicant (which matched the gender of the client). Participants read the following job description.

*Personal care aide*

*The work consists mainly of helping the client with daily tasks. The client is a ten-year-old [girl/boy] who lacks the ability to speak and can only communicate through simple gestures. The [girl/boy] has a condition which causes [her/his] ability to move to be limited and [she/he] needs help with things like eating, personal hygiene and transportation when [her/his] mother is at work (shift work). The person applying for the position should have worked as a personal care aide before or have similar experience. The person should be at least 20 years old. Particularly important for this position is to have a clear and trustworthy moral compass, so that the client is well taken care of despite [her/his] difficulty to communicate.*

Participants then received the following information about the applicant.

*Gender: [Woman/Man]*

*Age: 23*

*Previous work experience: Says that [she/he] has had a summer job as a shop assistant, worked in a kiosk for 3 years, and as a personal care aide for two years. Certificates of employment exist for all three positions.*

*Education: High school diploma with a specialization in childcare and sports.*

*Other info from the interview: Gives a serious impression and has charisma. Participates in a [Christian/Muslim/atheist/philosophical] discussion group in [her/his] spare time.*

*Reference: Has talked to the manager from the kiosk, who doesn’t say much. It seems like she doesn’t remember the person that well. The second reference, from the position as personal care aide, has not responded yet.*

After having read this information, participants rated the applicant on the seven 7-point Likert scale items regarding competence and likeability.

**Studies 3 and 4**

Participants first received two practice cases with applicants for a job as cleaner, which both had the same job description as in studies 1 and 2 for the first practice case. After the two practice cases, participants received two cases which both had the same job description. The two applicants both applied for a job as either personal care aide, teacher or administrative director of a municipality’s unit for economic support for people in need. In study 4, the last job was instead a regional commissioner at the Social Security Administration. To clarify, participants rated either two applicants for a position as personal care aide, two applicants for a position as teacher or two applicants for a position as administrative director/regional commissioner. All participants rated four applicants in total, including the practice cases.

***Practice Cases: Cleaner***

Participants read the following job description.

*Cleaner*

*This job requires experience with cleaning work. The person should autonomously clean offices in the building two days a week, which is expected to take 16 hours altogether. Important personal qualities for the job are social competence and consideration, since the cleaner will work while other people are working in the offices.*

Participants then received the following information about the first applicant.

*Applicant*

*Gender: Woman*

*Age: 37*

*Previous work experience: Has worked as a cleaner for about 17 years, and spent a couple of years with administrative work.*

*Education: Courses in cleaning at Helge’s Cleaning Inc. and Cleaning Service Inc., equivalent to 10 weeks.*

*Other info from the interview: The applicant gives a very confident impression. Seems committed and interested in the position. Judging from her answers on the interview questions, as well as the general impression during the interview, the applicant seems to function very well socially.*

*Reference: The reference is a manager at Cleaning Service Inc. who reports that he is very pleased with the applicant’s work. The applicant has worked in offices before and is used to being considerate to people working while she is cleaning.*

Participants then rated the applicant on the seven 7-point Likert scale items regarding competence and likeability. On the next page, the same job description was presented again, before the following information about the second applicant was given.

*Applicant*

*Gender: Woman*

*Age: 35*

*Previous work experience: The applicant has ample experience with cleaning both offices and other buildings. She has also worked in elderly care for a couple of years.*

*Education: Courses in cleaning at Cleaning Service Inc. and Office Cleaning, equivalent to about 12 weeks.*

*Other info from the interview: The applicant exudes energy and positivity. It is clear that she is interested in the position. During the whole interview she was pleasant, seemed to feel comfortable and answered the questions confidently.*

*Reference: The reference is the applicant’s current boss, who has many good things to say about her and nothing negative. In that position as well, the applicant is cleaning offices.*

Participants then rated the applicant on the seven 7-point Likert scale items regarding competence and likeability. They were not asked to choose which of the applicants they thought would be hired.

***Personal Care Aide***

These two cases were presented to a third of participants. Participants were either told about the applicant’s religious affiliation (target: Christian, Muslim or atheist) in the third case or in the fourth case. This was counterbalanced, as was gender of the applicant (which matched the gender of the client). The order of the two applicants presented below was also counterbalanced. Participants read the following job description.

*The work consists mainly of helping the client with daily tasks. The client is a ten-year-old [girl/boy] who lacks the ability to speak and can only communicate through simple gestures. The [girl/boy] has a condition which causes [her/his] ability to move to be limited, and [she/he] needs help with things like eating, personal hygiene and transportation when [her/his] mother is at work (shift work). The person applying for the position should have worked as a personal care aide before or have similar experience. The person should be at least 20 years old. Particularly important for this position is to have a clear and trustworthy moral compass, so that the client is well taken care of despite [her/his] difficulty to communicate.*

Participants then received information about one of the applicants (counterbalanced).

*Applicant*

*Gender: [Woman/Man]*

*Age: 32*

*Previous work experience: The applicant has done administrative work for a few years, been a [waitress/waiter] for an additional few years, and has worked as a personal care aide during the last 3-4 years.*

*Education: High school diploma with a specialization in hotel and catering service.*

*Other info from the interview: The applicant seems interested in the position. [She/He] seems to have much energy and says that [she/he] enjoys spreading happiness to the people [she/he] helps. Participates in a [Christian/Muslim/atheist/philosophical] discussion group in [her/his] spare time.*

*Reference: Talked to a manager from a restaurant, who doesn’t say much. It seems like she doesn’t remember the person that well. The second reference, from the personal care aide position, has not yet replied.*

Participants then rated the applicant on the seven 7-point Likert scale items regarding competence and likeability. On the next page, the same job description was presented again, before information about the next applicant was given (counterbalanced).

*Gender: [Woman/Man]*

*Age: 31*

*Previous work experience: The applicant has been a cold-buffet manager for several years, case worker at a company for an additional few years, and has now been a personal care aide for almost four years.*

*Education: The applicant has a high school diploma with a specialization in hotel and catering service.*

*Other info from the interview: The applicant seems to have a strong driving force and expresses that [she/he] is happy in the role as personal care aide. [She/He] says that [she/he] feels satisfied with her work when [she/he] can get the one [she/he] is aiding to laugh. Says that [she/he] is active in a [Christian/Muslim/atheist/interest] organization in [her/his] spare time.*

*Reference: Boss for position as personal care aide will call tomorrow. The boss at the company where the applicant was a case worker talks vaguely about the applicant, but at least does not mention anything negative.*

At the next page, participants saw the following text.

*Below, two applicants for the position as personal care aide are shown, both of which you have read about earlier in the study. Which one of these applicants would you hire?*

The job description was presented again, as well as the information about both applicants, presented side by side below the job description and labelled *Applicant A* and *Applicant B*. At the bottom of the page, the following question was asked.

*Which one of these applicants would you hire?*

*1. Applicant A*

*2. Applicant B*

***Teacher***

These two cases were presented to a third of participants. Participants were either told about the applicant’s religious affiliation (target: Christian, Muslim or atheist) in the third case or in the fourth case. This was counterbalanced, as was gender of the applicant. The order of the two applicants presented below was also counterbalanced. Participants read the following job description.

*Teacher grades 1-6*

*This position for a teacher at an elementary school entails that the person teaches one class from first to sixth grade in most school subjects. The person being hired should be a certified teacher. It is also important to have experience working with children and being able to handle children in the typical age span (6-12 years old) in a good way. This implies, for example, being able to stimulate the students’ appetite for learning, to identify and act in accordance with each student’s individual needs, as well as solve conflicts with students and between students. The position includes work with digital tools.*

Participants then received information about one of the applicants (counterbalanced).

*Applicant*

*Gender: [Woman/Man]*

*Age: 34*

*Previous work experience: Has worked as a teacher in a primary school (grades 1-3) for four years and has taught 5th graders at a larger school for almost a year. Before college, [she/he] worked as a storekeeper.*

*Education: Certified teacher for kindergarten and grades 1-3 (Michigan State University). Has also complemented [her/his] education with courses to gain qualification for teaching grades 4-6, but has some courses left to take.*

*Other info from the interview: The applicant is well-spoken and seems to be a social and observant person. Participates in a [Christian/Muslim/atheist/philosophical] discussion group in [her/his] spare time.*

*Reference: The reference is a principal at the applicant’s previous workplace, who had good experiences with the person.*

Participants then rated the applicant on the seven 7-point Likert scale items regarding competence and likeability. On the next page, the same job description was presented again, before information about the next applicant was given (counterbalanced).

*Applicant*

*Gender: [Woman/Man]*

*Age: 32*

*Previous work experience: The applicant has worked as a teacher in a primary school (grades 1-3) for five years and has during this time often substituted for teachers in grades 4-6. Has also previously been a delivery van driver.*

*Education: Certified teacher for kindergarten and grades 1-3 (Minnesota State University Moorhead), and has taken the larger parts of complementary courses needed for being a certified teacher for grades 4-6.*

*Other info from the interview: The applicant gives a social and relaxed impression, but answers the questions seriously. Says that [she/he] is active in a [Christian/Muslim/atheist/interest] organization in [her/his] spare time.*

*Reference: The reference is the principal at the current workplace and has some positive things to say about the applicant.*

At the next page, participants saw the following text.

*Below, two applicants for the position as teacher are shown, both of which you have read about earlier in the study. Which one of these applicants would you hire?*

The job description was presented again, as well as the information about both applicants, presented side by side below the job description and labelled *Applicant A* and *Applicant B*. At the bottom of the page, the following question was asked.

*Which one of these applicants would you hire?*

*1. Applicant A*

*2. Applicant B*

***Regional Commissioner***

These two cases were presented to a third of participants. Participants were either told about the applicant’s religious affiliation (target: Christian, Muslim or atheist) in the third case or in the fourth case. This was counterbalanced, as was gender of the applicant. The order of the two applicants presented below was also counterbalanced. Participants read the following job description.

*Regional commissioner*

*The Social Security Administration is seeking a regional commissioner. The person who will be hired will be responsible for the budget concerning the region’s social security interventions. The regional commissioner directly oversees the field site directors, who implement the decisions made by the regional commissioner. The position also involves recruitment of employees and operational management. This entails that the person should be a skilled leader, as well as being able to find creative solutions to problems and improvements in the regional organization. The position implies a large responsibility, as decisions being made will affect many people who benefit from social security in the region.*

Participants then received information about one of the applicants (counterbalanced).

*Applicant*

*Gender: [Woman/Man]*

*Age: 56*

*Previous work experience: The applicant has a long history of working within the government, for instance as a manager in elderly care, but has not previously supervised people in leading positions. [She/He] has also worked as an economist in the private sector.*

*Education: Master of Science in Business and Economics.*

*Other info from the interview: The applicant appears calm and friendly, makes a confident and interested impression. The applicant presented several ideas when asked how the regional organization can be improved. Participates in a [Christian/Muslim/atheist/philosophical] discussion group in [her/his] spare time.*

*Reference: The reference is the applicant’s current manager, who has good experiences with the person and believes that [she/he] would manage a role entailing greater responsibility well.*

Participants then rated the applicant on the seven 7-point Likert scale items regarding competence and likeability. On the next page, the same job description was presented again, before information about the next applicant was given (counterbalanced).

*Applicant*

*Gender: [Woman/Man]*

*Age: 55*

*Previous work experience: [She/he] started [her/his] career as an economist at a food business, but has worked for the government for the last fifteen years. The applicant has been a coordinator for home care service in the past, but has not had people in leading positions below [her/him] in previous positions.*

*Education: Master of Science in Business and Economics.*

*Other info from the interview: The applicant gives a likeable impression and answered all questions thoroughly. [She/He] also enthusiastically proposed ways in which the regional organization can be improved. Says that [she/he] is active in a [Christian/Muslim/atheist/interest] organization in [her/his] spare time.*

*Reference: A boss from a previous position has many positive things to say about the applicant. The role as regional commissioner would imply a larger responsibility than the applicant has previously had, but the reference thinks that the position would fit the applicant well.*

At the next page, participants saw the following text.

*Below, two applicants for the position as regional commissioner are shown, both of which you have read about earlier in the study. Which one of these applicants would you hire?*

The job description was presented again, as well as the information about both applicants, presented side by side below the job description and labelled *Applicant A* and *Applicant B*. At the bottom of the page, the following question was asked.

*Which one of these applicants would you hire?*

*1. Applicant A*

*2. Applicant B*

**Attention Check**

Participants were given the question below in order to show that they were paying attention to the instructions. Those who failed to follow the instructions were excluded from all analyses. In studies 1 and 2, the following attention check was used.

*Here is a different type of question. SKIP THE NEXT QUESTION. It is only included to ensure that you are paying attention and reading directions. Do not leave any answer on the question about number of applicants.*

*How many applicants have you evaluated in this study?*

*□ Two applicants*

*□ Three applicants*

*□ Four applicants*

In studies 3 and 4, the following attention check was used.

*Here is a different type of question. SELECT THE SECOND ALTERNATIVE ON THE QUESTION BELOW. It is only included to ensure that you are paying attention and reading directions. Therefore, select the second alternative below, “Three applicants”.*

*How many applicants have you evaluated in this study?*

*□ Two applicants*

*□ Three applicants*

*□ Four applicants*

**Purpose of the Study**

Participants then answered the question below. The purpose of this item was to control how many participants guessed the purpose of the study correctly. In study 1, 3.3% guessed correctly, in study 2, 4.2% guessed correctly, in study 3, 12.9% guessed correctly and in study 4, 10.9% guessed correctly. The correct answer was people’s perceptions about religious groups. These participants were not excluded.

*What do you think this study is mainly about so far?*

*□ Judgements with insufficient information*

*□ People’s perceptions about women and men*

*□ Recruiters’ decision processes*

*□ People’s perceptions about religious groups*

*□ Cooperation in recruitment processes*

**Demographic and Attitude Measures**

Below are all of the demographic questions as well as questions about participants’ religious beliefs and ideological beliefs. These were asked at the end of the study and participants were not able to go back to previous pages and change their answers.

*How old are you?*

*[Box to write in]*

*What is your gender?*

*□ Male*

*□ Female*

*□ Other*

*How strongly do you believe in God or gods (from 0-100)? To clarify, if you are certain that God (or gods) does not exist, please put "0" and if you are certain that God (or gods) does exist, then put "100."*

*[Box to write in]*

*What is your religious affiliation?*

*□ Christian (Catholic)*

*□ Christian (Baptist)*

*□ Christian (Other)*

*□ Hindu*

*□ Buddhist*

*□ Muslim*

*□ Jewish*

*□ Sikh*

*□ Believe in higher powers but no organized religion*

*□ Has not decided*

*□ Agnostic*

*□ None*

*□ Atheist*

*□ Other [box to write in]*

*Answer the question about how big of a role your religion plays in your life.*

*How big of a role does religion play in your life?*

*[Rating scale 1= none at all; 7=very central role]*

*We are interested in how you perceive your life. Think of a ladder representing where people stand in [insert country here]. At the top of the ladder are the people who are the best off – those who have the most money, the most education, and the most respected jobs. At the bottom are the people who are the worst off – who have the least money, least education, and the least respected jobs or no job. The higher up you are on this ladder, the closer you are to the people at the very top; the lower you are, the closer you are to the people at the very bottom. Imagine this rating scale represents the ladder. Where would you place yourself, relative to other people in USA?*

*[Rating scale from 0 (Bottom) to 10 (Top)]*

In study 1 and 3, the question above ended with “relative to other people in Sweden”. The question about location below was not asked in study 1 and 3.

*Location: City [Box to write in] State/Province [Box to write in]*

In study 1 and 3, the word “USA” was replaced with “Sweden” in the question below about national background.

*What is your national background?*

* Born in USA, with both parents born in USA*

* Born in USA, one parent born in USA*

* Born in USA, no parent born in USA*

* Born abroad, at least one parent born in USA*

* Born abroad, no parent born in USA*

*What is the highest degree of education you have completed?*

* Some high school*

* Completed high school or equivalent*

* Some university/college*

* Bachelor’s degree or equivalent*

* Master’s degree or equivalent*

* Some doctoral studies*

* PhD or equivalent*

*A number of organizations and other groups are listed below. Can you rate your trust in each of them? (1 = No trust at all; 10 = Very great trust)*

*• Police [Rating scale from 1 to 10]*

*• Courts [Rating scale from 1 to 10]*

*• The state [Rating scale from 1 to 10]*

*• People in general [Rating scale from 1 to 10]*

*The two questions below refer to your political opinions.*

*Where would you put yourself on the scale below, if 1 is complete financial safety (high taxes) and 10 is complete financial freedom (low taxes)?*

*[Rating scale from 1 to 10]*

*Where would you put yourself on the scale below, if 1 is to develop or change society as rapidly as possible and 10 is to preserve or maintain traditional ideas to the highest possible degree?*

*[Rating scale from 1 to 10]*
