# Supplementary material for: Religious affiliation seldom seems to influence hiring or competence ratings of job applicants: studies conducted in Sweden and in the USA
Source: BMC Psychol. 2022 Sep 19;10:220. doi: 10.1186/s40359-022-00927-0 (PMC9484152; doi:10.1186/s40359-022-00927-0)
Supplement: Supplementary file 2 — Additional file 2. Additional analyses. [file 40359_2022_927_MOESM2_ESM.docx]

Additional Analyses

In Table 1 below, the means and standard deviations of two measures of religiosity is shown for each study and each religious group. The *Belief in God* measure was obtained by asking the following question:

*How strongly do you believe in God or gods (from 0-100)? To clarify, if you are certain that God (or gods) does not exist, please put "0" and if you are certain that God (or gods) does exist, then put "100."*

The *Role of religion in life* question was asked in the following way:

*Answer the question about how big of a role your religion plays in your life.*

*How big of a role does religion play in your life?*

*[Rating scale 1= none at all; 7=very central role]*

| **Table 1**  *Religiosity measures of each religious group in each study* | | | | | |
| --- | --- | --- | --- | --- | --- |
|  |  | Atheist/None | Agnostic, etc. | Christian | Total |
|  |  | *M* (*SD*) | *M* (*SD*) | *M* (*SD*) | *M* (*SD*) |
| Belief in God | Study 1 | 1.19 (4.49) | 22.29 (29.97) | 23.50 (30.02) | 10.87 (22.57) |
|  | Study 2 | 9.49 (22.98) | 23.06 (26.04) | 84.47 (25.09) | 52.94 (42.04) |
|  | Study 3 | 3.13 (8.58) | 17.82 (21.90) | 47.00 (38.17) | 17.43 (28.69) |
|  | Study 4 | 11.46 (24.55) | 31.19 (31.69) | 83.28 (24.51) | 57.01 (40.39) |
|  | All studies | 6.19 (17.25) | 23.87 (27.37) | 79.05 (29.66) | 42.45 (41.53) |
| Role of religion in life | Study 1 | 1.44 (1.16) | 2.35 (1.54) | 2.40 (1.58) | 1.85 (1.40) |
|  | Study 2 | 1.27 (0.93) | 1.46 (0.80) | 4.97 (1.76) | 3.31 (2.27) |
|  | Study 3 | 1.29 (0.82) | 1.78 (1.10) | 3.24 (2.04) | 1.89 (1.52) |
|  | Study 4 | 1.29 (0.77) | 1.81 (1.19) | 4.99 (1.68) | 3.55 (2.22) |
|  | All studies | 1.30 (0.87) | 1.72 (1.09) | 4.76 (1.85) | 2.92 (2.16) |

A one-way ANOVA of belief in God showed that atheists, agnostics and Christians differed significantly in all studies (all *p* < .001), with the exception of agnostics and Christians in study 1, who did not differ significantly. Christians believed more strongly in God than agnostics and atheists and agnostics believed more strongly in God than atheists.

A one-way ANOVA of role of religion in life showed no significant differences in study 1, but significant differences between atheists, agnostics and Christians in studies 2-4, except atheists and agnostics in study 2, who did not differ significantly. Religion had a larger role in the lives of Christians than in the lives of agnostics and atheists (all *p* < .001) and a larger role in the lives of agnostics than in the lives of atheists in study 3 (*p* = .002) and in study 4 (*p* = .042).
